# Feasibility of Early Infant Diagnosis of HIV in Resource-Limited Settings: The ANRS 12140-PEDIACAM Study in Cameroon

**DOI:** 10.1371/journal.pone.0021840

**Published:** 2011-07-19

**Authors:** Mathurin C. Tejiokem, Albert Faye, Ida C. Penda, Georgette Guemkam, Francis Ateba Ndongo, Gisèle Chewa, Claire Rekacewicz, Dominique Rousset, Anfumbom Kfutwah, Pascal Boisier, Josiane Warszawski, the ARNS 12140-PEDIACAM study group

**Affiliations:** 1 Centre Pasteur du Cameroun, Membre du Réseau International des Instituts Pasteur, Yaoundé, Cameroun; 2 Université de Paris Sud 11, Paris, France; 3 Assistance Publique des Hôpitaux de Paris, Service de Pédiatrie Générale, Hôpital Robert Debré, Paris, France; 4 Université Paris Diderot, Sorbonne Paris Cité, UFR de Médecine, Paris, France; 5 Hôpital Laquintinie, Douala, Cameroun; 6 Université de Douala, Douala, Cameroun; 7 Centre Mère et Enfant de la Fondation Chantal Biya, Yaoundé, Cameroun; 8 Centre Hospitalier d'Essos, Yaoundé, Cameroun; 9 Agence Nationale de Recherches sur le Sida et les Hépatites Virales, Paris, France; 10 Institut Pasteur de Paris, Paris, France; 11 Assistance Publique des Hôpitaux de Paris, Service d'Epidémiologie et de Santé Publique, Hôpital de Bicêtre, Paris, France; 12 Equipe 4 (VIH et IST) - INSERM U1018 (CESP), Paris, France; University of Cape Town, South Africa

## Abstract

**Background:**

Early infant diagnosis (EID) of HIV is a key-point for the implementation of early HAART, associated with lower mortality in HIV-infected infants. We evaluated the EID process of HIV according to national recommendations, in urban areas of Cameroon.

**Methods/Findings:**

The ANRS12140-Pediacam study is a multisite cohort in which infants born to HIV-infected mothers were included before the 8^th^ day of life and followed. Collection of samples for HIV DNA/RNA-PCR was planned at 6 weeks together with routine vaccination. The HIV test result was expected to be available at 10 weeks. A positive or indeterminate test result was confirmed by a second test on a different sample. Systematic HAART was offered to HIV-infected infants identified. The EID process was considered complete if infants were tested and HIV results provided to mothers/family before 7 months of age. During 2007–2009, 1587 mother-infant pairs were included in three referral hospitals; most infants (n = 1423, 89.7%) were tested for HIV, at a median age of 1.5 months (IQR, 1.4–1.6). Among them, 51 (3.6%) were HIV-infected. Overall, 1331 (83.9%) completed the process by returning for the result before 7 months (median age: 2.5 months (IQR, 2.4–3.0)). Incomplete process, that is test not performed, or result of test not provided or provided late to the family, was independently associated with late HIV diagnosis during pregnancy (adjusted odds ratio (aOR) = 1.8, 95%CI: 1.1 to 2.9, p = 0.01), absence of PMTCT prophylaxis (aOR = 2.4, 95%CI: 1.4 to 4.3, p = 0.002), and emergency caesarean section (aOR = 2.5, 95%CI: 1.5 to 4.3, p = 0.001).

**Conclusions:**

In urban areas of Cameroon, HIV-infected women diagnosed sufficiently early during pregnancy opt to benefit from EID whatever their socio-economic, marital or disclosure status. Reduction of non optimal diagnosis process should focus on women with late HIV diagnosis during pregnancy especially if they did not receive any PMTCT, or if complications occurred at delivery.

## Introduction

By the end of 2009, an estimated 2.5 million children worldwide were living with HIV, mostly a consequence of vertical transmission, and more than 90% of these children are in sub-Saharan Africa [1]. In resource limited countries, about 35% of HIV infected infants without any therapeutic intervention die before the age of one year [2]. Observational studies in industrialized countries and a randomized clinical trial in South Africa demonstrated that early initiation of HAART in infants dramatically reduces morbidity and mortality [3]–[6]. These results led the World Health Organization (WHO), in 2008, to recommend systematic early initiation of HAART to all HIV-infected infants diagnosed within the first year of life, and since 2010, within the first two years of life, irrespective of CD4 count or WHO clinical stage [7], [8]. Thus, early diagnosis of HIV infection in infants is essential. In 2008, only 15% of HIV-exposed infants benefited from recommended virological tests within the first two months of life [9].

Many sub-Saharan African countries are increasingly developing and scaling up access to early infant HIV virological testing, particularly using dried blood spots (DBS) [9], [10], [11] generally collected in clinical sites and sent to reference laboratories. In Cameroon a pilot program of HIV early infant diagnosis, was launched in 2007 using DBS with clinical sites collecting and sending samples to two referral laboratories for testing. The main difficulties encountered were the long delay between blood collection and the result, the large proportion of parents who failed to return for their infants' results and HIV-infected infants being lost to follow-up [12]. Several studies on PMTCT programs and clinical cohorts throughout sub-Saharan Africa similarly experienced high rates of loss to follow up [13]–[15] and some identified poor socioeconomic conditions as being a cause [14], [16], [17]. Despite the investment of financial and human resources, it remains of primary importance to improve the acceptability of early HIV testing in infants.

The ANRS 12140-PEDIACAM study started in Cameroon in November 2007 with two main objectives: to study the feasibility and effectiveness, in routine practice, of early antiretroviral multitherapy offered systematically to HIV-infected infants before 7 months of age; and to evaluate the humoral response of these children to vaccines of the Expanded Program of Immunization (EPI). All infants born to HIV-infected mothers included in the Pediacam study were offered early infant diagnosis (EID). Here, we describe the complete EID process (HIV test performed and results provided to mothers/family before 7 months of age) in urban areas of Cameroon and identify maternal and infant characteristics associated with the EID process being incomplete.

## Methods

### The ANRS 12140-Pediacam survey

Pediacam is an ongoing prospective observational cohort coordinated at the Centre Pasteur of Cameroon, and conducted in three referral hospitals in the two largest cities of Cameroon: the Central Hospital Maternity (CHM)/Mother and Child Center of the Chantal Biya Foundation (MCC-CBF), the Hospital Center Essos (HCE) both in Yaounde and Laquintinie Hospital (LH) in Douala. These referral hospitals are among the pioneers in the fight against HIV infection in Cameroon and offer a full range of services for infant and adult health needs in their areas. The Pediacam study comprises two consecutive phases. All infants born live to HIV-infected mothers with documented serostatus were included before the 8^th^ day of life in the first phase of the study, and were followed, tested for HIV and given routine vaccinations by nurses according to the Cameroonian Expanded Program on Immunization schedule: BCG and oral polio vaccine at birth, combination diphtheria, tetanus, pertussis, hepatitis B vaccine and *Haemophilus influenzae* type b (Hib) vaccine, oral polio vaccine at 6, 10 and 14 weeks. Prior to inclusion, mothers were provided, by the physicians involved in the study, with information about the study, the benefits of follow up and infant testing at 6 weeks, and were invited to give written informed consent for the participation of their infant in the study. During the first visit planned at 6 weeks, early HIV diagnosis was proposed after counselling in addition to the routine clinical, nutritional and immunization follow up. In the second phase, systematic HAART and follow up was offered to all HIV-infected children included in the first phase, and also to HIV-infected children not followed since birth but aged less than 7 months at diagnosis of HIV infection. Infants born to HIV-uninfected mothers were also included in a control group. The ANRS-Pediacam study was granted ethical approval in Cameroon by the National Ethics Committee and in France by the Biomedical Research Committee of the Pasteur Institute of Paris. The Cameroon Ministry of Public Health gave administrative authorization to start the study.

### Study population

This analysis included all infants born live to HIV-infected mothers enrolled into the first phase of Pediacam from November 2007 to December 2009. They were all aged at least six months at the time of this analysis.

### Process of early infant diagnosis of HIV

Infant follow-up was scheduled to coincide with the Cameroon National EPI timetable at 6, 10 and 14 weeks thereby limiting the number of hospital visits. Samples for HIV virological testing were collected from HIV-exposed infants at the first follow-up visit planned at 6 weeks of age and the results were expected to be available at the next visit planned at 10 weeks. The study physicians and psychosocial workers contributed to post-test counselling. A positive or indeterminate test result was confirmed by a second test on a different sample collected as soon as possible after a phone call to the parents. A second test was also planned for breastfed infants, six weeks after weaning. In the Pediacam study, information concerning infant feeding was reported orally by mothers at inclusion i.e. before the 8^th^ day of life and during the first phase follow up visits at 6, 10 and 14 weeks. The central coordinating team regularly identified infants who did not return for a follow-up visit within one week of the planned date and organised with the clinical site to make reminder phone calls. Incentives, including free medical support for consultation, HIV diagnosis, additional vaccine (*Haemophilus influenzae* type b which was not included in the Expanded Program on Immunization at the time this project was launched), and reimbursement of taxi fares were provided to participants by the Pediacam study during follow-up visits. The project provided free milk as required to women who choose formula feeding. For infants who tested HIV negative but were not included in the second phase of the Pediacam study, clinical follow up was encouraged until at least the HIV serological test at 15–18 months, in line with the national recommendations. The EID process from birth to the announcement of the result to the family was considered to be “complete” if the HIV virological test was performed and the result provided to mothers/family before 7 months of age.

### Virological determination of HIV status

At the first follow-up visit for HIV-exposed infants attending the Yaoundé sites, whole blood was collected into EDTA-containing anticoagulant tubes and transferred to the Virology Laboratory of Centre Pasteur of Cameroon. The samples were tested for HIV RNA by a real-time polymerase chain reaction (RT PCR) (Biocentric HIV Charge Virale) using the TaqMan technology in an in-house protocol validated by the French National Agency for Research on AIDS and Viral Hepatitis [18]. Samples from infants attending the Douala site were collected onto filter papers (DBS), processed according to standard procedures [19] and transported to the National Program Reference laboratory, managed by the US Center for Disease Control at Mutengene. To reduce the delay between blood collection and availability of the test result at the collection site, one of the DBS collected from each infant at the Douala site was also sent to the Virology Laboratory of Centre Pasteur of Cameroon. Biocentric HIV DNA cell kit was used to test for HIV proviral DNA. All samples were also subjected to an in-house serotyping assay to distinguish between HIV groups and indirectly confirmed maternal HIV serological status.

### Data collection

Data concerning socio-demographic and obstetrical characteristics, strategies for prevention of mother-to-child transmission, neonatal anthropometric and clinical characteristics, postnatal treatment, feeding option, and routine immunization were collected in standardized questionnaires for each mother-child pair included. Clinical, vaccination and nutrition status was evaluated at each visit.

### Statistical analysis

Characteristics of mother-infant pairs were described using medians with interquartile ranges (IQR) for continuous variables and percentages with frequencies for categorical variables. Each step of the EID process was described, and proportions were estimated with 95% confidence intervals (95%CI). For multiple births, only one infant, randomly selected was included in the statistical analysis. Univariate and multivariate logistic regressions were performed to identify factors associated with “incomplete EID process”.

A first multivariate logistic regression model included non collinear variables associated with “incomplete EID process” (as dependant variable) if the p-value was ≤0.2 in the univariate analysis. The final model was obtained using a backward-stepwise strategy with consideration for confounding effects and interactions. We systematically included the study site and the mother's education level. Statistical analyses were performed using STATA version 10.0 (Stata Corp, College Station, TX, USA). A p-value of 5% was considered for statistical significance of association.

## Results

### Baseline characteristics of the study population

Between November 2007 and December 2009, 1645 infants born to 1587 HIV infected mothers at three referral hospitals were included in the ANRS 12140-Pediacam study: 756 (45.9%) at the CH/MCC-CBF; 406 (24.7%) at the HCE in Yaoundé and 483 (29.4%) at the LH in Douala. Before pregnancy, 666 (42.0%) of the 1587 mothers were aware of their HIV infection. The median age of mothers at delivery was 29.2 years (IQR, 25.8–32.8). Among the mothers, 89.2% delivered vaginally, 3.5% had multiple births (52 twins and four triplets; one infant each died at delivery in the case of two twins), 9.9% did not take any prophylaxis for PMTCT, and 10.7% declared breastfeeding exclusively. Of the mother-infant pairs enrolled, 1176 (74.1%) mothers delivered in maternities participating in the study, 392 (24.7%) in other maternities, most of which were located in the same cities as the referral hospitals, and 19 (1.2%) at home or on their way to health facilities to deliver. The median age at enrolment of the HIV-exposed infants was 3 days (IQR, 1–5) and 51.8% were male. The median birth weight was 3150 g (IQR, 2800–3500).

### Early infant diagnosis process

Of the 1587 mother-infant pairs, 90.2% (1431) attended at least one scheduled follow-up visit. Only 0.6% (8) of the infants were not tested, although the mothers agreed to HIV testing during the consultation. The mothers of 94.9% (1351) of 1423 infants tested returned to collect the result: 75.3% (1018) received the result at or before the infant was 3 months old; 23.2% (313) between ages 4 and 6 months; and only 1.5% (20) were tested and learned about their HIV result after 6 months of age. The median age at HIV testing was 1.5 months (IQR, 1.4–1.6), with no significant differences observed between the sites, and at delivery of the result was 2.5 months (IQR, 2.4–3.0). The median time between HIV testing and receipt of the test result was 1.1 month (IQR, 1.0–1.2) with statistically significant difference according to site of inclusion (1.0 month for CH/MCC-CBF, 1.0 month for HCE and 1.5 month for LH ; p<10^−3^). HIV infection was diagnosed in 3.7% (95%CI: 2.8–4.8, n = 50) of infants whose family returned to collect result. Of the 5.1% (n = 72) of infants whose parents did not return to learn about their HIV result, only one had a positive HIV PCR result, and had died before the test result could be returned.

**Figure 1:**
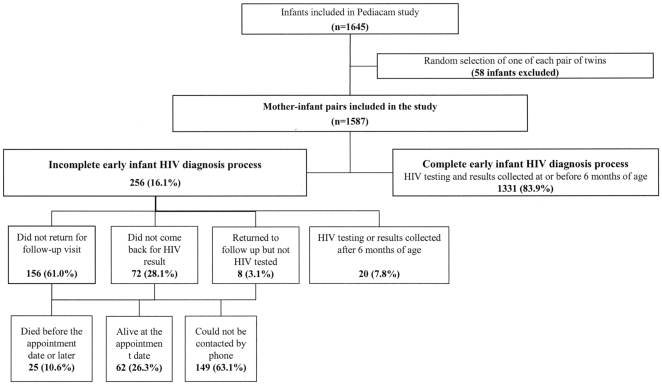
Take-up and key points of early infant HIV diagnosis process (ANRS12140-Pediacam study, Cameroon, 2007–2009).

Overall, the EID process was incomplete for 16.1% (95%CI: 14.4–18.0; n = 256) of the 1587 HIV-exposed infants: these infants were either not tested or their families did not return to collect the HIV results before 7 months of age (Fig. 1). The vital status of infants who did not return for a follow-up visit was determined by phone calls in 87 cases: 25 died before HIV testing or collection of results, including 14 deaths before the first visit scheduled before 7 weeks of age. Of the parents of the 62 remaining infants, 45 gave various reasons to justify their failure to attend the follow up or to collect the HIV result. Over half (57.8%) reported moving and in 11.1%, the mothers were sick. Other reasons included refusal of HIV testing, fear of the HIV test results, forgetting about the follow up visit, the time required for the consultation, attending follow up in another health structure, and death of a parent. For 149 infants, no information could be obtained concerning the vital status because they could not be traced mostly due to missing or incorrect contact information (Fig. 1). These infants were considered to be lost to follow up.

### Factors associated with incomplete early infant diagnosis process

In univariate analysis, “incomplete EID process” was significantly associated with late HIV diagnosis during pregnancy (less than 3 months before delivery), few antenatal visits (less than 4 visits), absence of MTCT prophylaxis, emergency caesarean section for delivery, low mother educational level, the lack of a functional refrigerator, and non disclosure of maternal HIV status to the partner (**Table S1**). “Incomplete EID process” also tended to be more frequent among cases of premature delivery (less than 37 weeks of pregnancy, p = 0.09), multiple birth (p = 0.07) and infant hospitalisation at birth (p = 0.07).

In multivariate analysis (**Table 1**), “incomplete EID process” remained independently associated with late HIV diagnosis during pregnancy (adjusted odds ratio (aOR): 1.8; 95%CI: 1.1 to 2.9; p = 0.01), absence of MTCT prophylaxis (aOR: 2.4; 95%CI: 1.4 to 4.3; p = 0.002), and emergency caesarean section (aOR: 2.5, 95%CI: 1.5 to 4.3; p = 0.001). Multiple birth tended to be associated (aOR  = 1.8, 95%CI: 0.9 to 3.7, p = 0.10). None of low mother's education level, the lack of a functional fridge, non disclosure of HIV status to partner, or few antenatal visits were associated with “incomplete EID process”.

**Table 1:** Multivariate analysis: factors associated with incomplete early infant HIV diagnosis process (ANRS12140-Pediacam study, Cameroon, 2007–2009).

	Initial logistic regression model		Final logisticregression model	
Total (n = 1285)	adjusted OR (CI 95%)	p-value	adjusted OR (CI 95%)	p-value
Clinical sites		0.25		0.33
EHC*	1.2 (0.8–1.8)	0.44	1.2 (0.8–1.8)	0.44
LH†	0.8 (0.5–1.2)	0.28	0.8 (0.5–1.2)	0.32
CHM/MCC-CBF‡	ref		ref	
Mothers' level of education		0.50		0.36
None or primary education	1.4 (0.8–2.5)	0.24	1.5 (0.8–2.6)	0.15
Secondary	1.2 (0.8–2.0)	0.40	1.3 (0.8–2.1)	0.29
Higher education	ref		ref	
Presence of a functional fridge at home				
No	1.2 (0.8–1.6)	0.33		
Yes	ref			
Time at maternal HIV diagnosis		0.10		0.04
Less than 3 months before delivery	1.7 (1.0–2.7)	0.04	1.8 (1.1–2.9)	0.01
More than 3 months before delivery	1.2 (0.8–1.7)	0.50	1.2 (0.8–1.8)	0.36
Before pregnancy	ref		ref	
Disclosure of maternal HIV serostatus to partner				
No	1.3 (0.8–1.9)	0.27		
Yes	ref			
Maternal knowledge of the fathers' HIV status		0.25		
Did not know	1.2 (0.7–1.9)	0.47		
Negative	1.4 (0.9–2.2)	0.11		
Positive	ref			
Number of antenatal visits				
≤3	1.1 (0.7–1.7)	0.72		
>3	ref			
Mode of delivery		0.005		0.003
Emergency C-section	2.5 (1.4–4.3)	0.001	2.5 (1.5–4.3)	0.001
Elective C-section	0.7 (0.2–2.2)	0.59	0.8 (0.3–2.2)	0.62
Vaginal	ref		ref	
Receipt of any ART for PMTCT		0.02		0.009
No prophylaxis	2.3 (1.2–4.1)	0.005	2.4 (1.4–4.3)	0.002
Short-course combined ARV	1.4 (0.9–2.1)	0.14	1.3 (0.9–2.0)	0.16
Tritherapy	ref		ref	
Birth weight				
<2500 g	1.3 (0.7–2.2)	0.38		
≥2500 g	ref			
Prematurity				
<37 weeks	1.0 (0.6–1.6)	0.92		
≥37 weeks	ref			
Multiple birth				
Yes	1.5 (0.7–3.4)	0.26	1.8 (0.9–3.7)	0.10
No	ref		ref	
Infant hospitalised at birth				
Yes	1.2 (0.7–1.9)	0.57		
No	ref			

*EHC : Essos Hospital Center, Yaoundé.

† LH : Laquintinie Hospital, Douala.

‡ CHM / MCC-CBF: Central Hospital Maternity / Mother and Child Center of the Chantal Biya Foundation, Yaoundé.

## Discussion

Early infant diagnosis of HIV is one of the most important challenges in the management of paediatric HIV infection in resource limited settings. Here, we describe each step and obstacles to EID in infants included in the ANRS 12140-PEDIACAM study. This study involves a large prospective cohort mainly designed to evaluate the feasibility of early HAART for HIV-infected infants at three reference hospitals in the two main cities in Cameroon. As far as we are aware, this is one of the first large prospective studies to identify factors associated with the process of infant HIV diagnosis being incomplete in African settings where early virological testing and antiretroviral drugs are now available for infants outside of the research context.

In this study, the EID process from birth to the announcement of the result to the family was considered as complete if the HIV result was provided to the family before the infant was 7 months old. Learning the HIV status of the child after age 6 months is too late to allow early initiation of HAART, in accordance with several studies [3], [5]. This would therefore result in a missed opportunity for HIV infected infants. Thus, the Pediacam study recommends starting treatment before age 7 months for all infants diagnosed as HIV-infected in the first phase of the study.

In the Pediacam study, 90.2% of infants returned at least once for follow up and almost all were tested for HIV. The return rate was higher than those previously reported in Malawi [14] and Uganda [20] where 70% (1503/2156) and 52% (292/567) of HIV exposed infants, respectively, were brought back for follow up. However, antiretroviral drugs were not easily accessible to children at the time of these previous surveys. Our active tracing method with multiple reminder calls may also explain the higher rate of return in our study.

Overall, the EID process was complete for 83.9% (95%CI: 82.0–85.6) of infants before the age of 7 months. This rate is similar to that reported in one clinical setting in Botswana (81%; 753/930) [11], but higher than those in surveys conducted in Tanzania and in South Africa where 55% (242/441) [21] and 65% (153/233) [22], respectively, of parents/guardians returned to learn about the results. However, age at diagnosis in these studies was more diverse: between 1 and 17 months. In the Pediacam study, the median age at collection of the HIV result was 2.5 months (IQR, 2.4–3.0). This is satisfactory in relation to promoting very wide access to HAART before 6 months of age. Initiating HAART at 3 months, as planned in the CHER randomized trial [4], would be feasible in routine practice for 75% of the infants in such settings.

Of every 100 infants offered early testing, 10 did not return for the test and six were tested but the family did not return to collect the result before the age of 7 months (both situations being incomplete EID). We investigated factors associated with incomplete EID. Late HIV diagnosis during pregnancy and absence of prophylaxis for PMTCT were significantly and independently associated with a higher risk of “incomplete EID process”. Loss to follow up was previously reported to be associated with no PMTCT prophylaxis in a study in Uganda [20], and with short time between HIV infection and inclusion in a study for adults in France [23]. Of the 157 women who did not receive any PMTCT prophylaxis in Pediacam, 65.0% were diagnosed at a median time of 3 days (IQR, 1 to 15 days) before delivery (result not showed). Such a short period does not allow the women time to cope with the result, to deal with issues around disclosure to partner and to family, and to appreciate fully the benefits of medical follow up for both her and her child [24].

Incomplete EID was also significantly and independently associated with emergency caesarean section. Incomplete EID also tended to be more frequent in cases of multiple birth, low birth weight and infant hospitalisation at birth. Multiple birth and caesarean section were not reported to be associated with loss to follow up in a study conducted in Malawi [14]. Although our finding of an association with emergency caesarean section was unexpected, there are at least two possible explanations. First, saving the life of the mother and infant is the primary concern in cases of obstetrical emergency or neonatal complications. In such situations, prevention of HIV transmission may be a lower immediate priority for the mother and the medical team, and there may be little opportunity to discuss the benefits of follow up and infant HIV testing. Second, complications of labour or delivery are likely to be one of the reasons to refer women followed in primary antenatal care setting to the hospitals participating in the Pediacam survey. These women may prefer to return after delivery to their local hospital for infant follow up. Unfortunately, the referral status of pregnant women was not collected in Pediacam study so it is not possible to test whether or not this was indeed the case.

Incomplete EID process was also associated, in univariate analysis, with factors related to living conditions and socio-economic status: low level of mother education, absence of a functional fridge at home, and failure to disclose their HIV status to partner. However, odds ratios were lower for these factors than for factors related to late HIV diagnosis and obstetrical context of delivery (**Table S1**). Moreover, these associations did not remain significant in multivariate analysis (**Table 1**). Our results thus contrast with those of other studies which report a higher rate of loss to follow up in poor socioeconomic conditions (low education level, farming, long distances and cost of transport) [14], [25] and in cases of non disclosure of HIV status [16].

The median time between HIV testing and receipt of test result differed between Pediacam sites, mainly due to the DBS being used in one of the sites. More time is needed for preparation, conditioning and transport of DBS to the laboratory. This did not affect the completeness of the process within 6 months, but could do so if the aim was to complete the process in 3 months. This underlines the importance of carefully evaluating local obstacles to implementation of an EID programme.

The proportion of mothers initiating formula feeding was higher than is generally the case nationwide, but was consistent with what has been described previously in these settings [26], [27]. In 2009, only 32% of HIV-infected mothers reported formula feeding [12]. The infant feeding policy in Cameroon promotes exclusive breastfeeding for the first six months, unless replacement feeding is acceptable, feasible, affordable, sustainable and safe. The high proportion of formula feeding in the Pediacam study may be explained by the active counselling, by the higher economic status and lower socio-cultural pressure on feeding practices in urban areas than in others areas in the country.

This study has some limitations. The inclusion of infants born to HIV-infected mothers at Pediacam study maternities was not exhaustive. The failure to return may in some cases have been the consequence of mortality. However, the vital status could not be established for 149 of the 256 children with incomplete process (**Fig. 1**). The mortality rate during the process, estimated among the 1438 infants with known vital status, was 17.4 per 1000 live births (95%CI: 11.3–25.6, n = 25), which is lower than the estimated national rate in Cameroon: 29‰ in the first month [28]. This lower rate may have been due to better early clinical management. The value, however, may also reflect a high rate of early mortality among the infants who never returned, possibly due to severe HIV infection. Indeed, continuing this argument would suggest that the transmission rate for HIV of 3.6% (2.8 – 4.8) may be an underestimation.

Despite these limitations, one of the strengths of Pediacam study was that it was conducted in three centres in two large cities of Cameroon. These centres have very different working practices and recruitments so the study reflects the diversity of practical management of EID in a heterogeneous population mostly living in urban areas.

In summary, our study shows good take-up of EID services and high proportion of mothers returning to collect their infant's result within the first six months of life in an operational setting. As nearly half of the mothers knew their HIV status before pregnancy, most of them were likely to have knowledge of the benefits of PMTCT, and infant HIV diagnosis and follow up. In the Pediacam study, the risk factors for “incomplete EID process” reflected the quality of prenatal care and the obstetrical context (late HIV diagnosis, lack of any prophylaxis for PMTCT, emergency caesarean section), rather than environmental conditions such as socio-economic, marital and disclosure status. This may be partly due to the follow-up schedule being tied to the national vaccination programme, and partly to incentives and active reminders by telephone calls.

It seems to be very important to focus on women diagnosed at, or only a few days before, delivery, especially if they did not receive any PMTCT, or if they were transferred from their usual care setting for emergency delivery. All maternity team members should be aware of these issues, so as to offer reinforced support just after delivery in these situations at risk of non optimal follow up of the infant.

## Supporting Information

Table S1 Univariate analysis: factors associated with incomplete HIV early infant diagnosis process: (ANRS 12140-Pediacam study, Cameroon, 2007–2009).
(DOC)

Appendix S1 The ANRS 12140-Pediacam Study Team.
(DOC)

## References

[1] UNAIDS (2010). UNAIDS report on the global AIDS epidemic 2010.. http://www.unaids.org/en/KnowledgeCentre/HIVData/EpiUpdate/EpiUpdArchive/2009/default.asp.

[2] Newell ML, Coovadia H, Cortina-Borja M, Rollins N, Gaillard P (2004). Mortality of infected and uninfected infants born to HIV-infected mothers in Africa: a pooled analysis.. Lancet.

[3] Faye A, Le Chenadec J, Dollfus C, Thuret I, Douard D (2004). Early versus deferred antiretroviral multidrug therapy in infants infected with HIV type 1.. Clin Infect Dis.

[4] Violari A, Cotton MF, Gibb DM, Babiker AG, Steyn J (2008). Early antiretroviral therapy and mortality among HIV-infected infants.. N Engl J Med.

[5] Chiappini E, Galli L, Tovo PA, Gabiano C, Lisi C (2009). Five year follow-up of children with perinatal HIV-1 infection receiving early highly active antiretroviral therapy.. BMC Infect Dis.

[6] Goetghebuer T, Haelterman E, Le Chenadec J, Dollfus C, Gibb D (2009). Effect of early antiretroviral therapy on the risk of AIDS/death in HIV-infected infants.. Aids.

[7] World Health Organization (WHO) (2008). Report of the WHO Technical Reference Group, Paediatric HIV/Antiretroviral Therapy and Care Guideline Group Meeting, WHO headquarters, Geneva, Switzerland, 10-11April2008.. http://www.who.int/hiv/pub/paediatric/WHO_Paediatric_ART_guideline_rev_mreport_2008.pdf.

[8] World Health Organization (WHO) (2010). Antiretroviral therapy for HIV infection in infants and children. Towards Universal Access. Recommendations for public health approach. 2010 revision.. http://whqlibdoc.who.int/publications/2010/9789241599801_eng.pdf.

[9] World Health Organization (WHO), UNICEF, UNAIDS (2010). Towards universal access: scaling up priority HIV/AIDS interventions in the health sector. Progress report 2009.. http://www.who.int/hiv/pub/tuapr_2009_en.pdf.

[10] Creek TL, Sherman GG, Nkengasong J, Lu L, Finkbeiner T (2007). Infant human immunodeficiency virus diagnosis in resource-limited settings: issues, technologies, and country experiences.. Am J Obstet Gynecol.

[11] Creek T, Tanuri A, Smith M, Seipone K, Smit M (2008). Early diagnosis of human immunodeficiency virus in infants using polymerase chain reaction on dried blood spots in Botswana's national program for prevention of mother-to-child transmission.. Pediatr Infect Dis J.

[12] Ministère de la Santé Publique du Cameroun, Comité National de Lutte contre le SIDA (2009). Vers l'accès universel à la prévention en faveur des groupes cibles prioritaires: prévention de la transmission du VIH de la mère à l'enfant. Rapport de progrès N°4.. http://www.cnls.org/public/web/IMG/pdf/rapport_ptme_n_4.pdf.

[13] Nyandiko WM, Otieno-Nyunya B, Musick B, Bucher-Yiannoutsos S, Akhaabi P (2010). Outcomes of HIV-exposed children in western Kenya: efficacy of prevention of mother to child transmission in a resource-constrained setting.. J Acquir Immune Defic Syndr.

[14] Ioannidis J, Taha T, Kumwenda N, Broadhead R, Mtimavalye L (1999). Predictors and impact of losses to follow-up in an HIV-1 perinatal transmission cohort in Malawi.. Int J Epidemiol.

[15] Manzi M, Zachariah R, Teck R, Buhendwa L, Kazima J (2005). High acceptability of voluntary counselling and HIV-testing but unacceptable loss to follow up in a prevention of mother-to-child HIV transmission programme in rural Malawi: scaling-up requires a different way of acting.. Trop Med Int Health.

[16] Jones SA, Sherman GG, Varga CA (2005). Exploring socio-economic conditions and poor follow-up rates of HIV-exposed infants in Johannesburg, South Africa.. AIDS Care.

[17] Bwirire LD, Fitzgerald M, Zachariah R, Chikafa V, Massaquoi M (2008). Reasons for loss to follow-up among mothers registered in a prevention-of-mother-to-child transmission program in rural Malawi.. Trans R Soc Trop Med Hyg.

[18] Rouet F, Ekouevi DK, Chaix ML, Burgard M, Inwoley A (2005). Transfer and evaluation of an automated, low-cost real-time reverse transcription-PCR test for diagnosis and monitoring of human immunodeficiency virus type 1 infection in a West African resource-limited setting.. J Clin Microbiol.

[19] Sherman GG, Cooper PA, Coovadia AH, Puren AJ, Jones SA (2005). Polymerase chain reaction for diagnosis of human immunodeficiency virus infection in infancy in low resource settings.. Pediatr Infect Dis J.

[20] Ahoua L, Ayikoru H, Gnauck K, Odaru G, Odar E (2010). Evaluation of a 5-year programme to prevent mother-to-child transmission of HIV infection in Northern Uganda.. J Trop Pediatr.

[21] Nuwagaba-Biribonwoha H, Werq-Semo B, Abdallah A, Cunningham A, Gamaliel JG (2010). Introducing a multi-site program for early diagnosis of HIV infection among HIV-exposed infants in Tanzania.. BMC Pediatr.

[22] Rollins N, Mzolo S, Moodley T, Esterhuizen T, van Rooyen H (2009). Universal HIV testing of infants at immunization clinics: an acceptable and feasible approach for early infant diagnosis in high HIV prevalence settings.. Aids.

[23] Lanoy E, Mary-Krause M, Tattevin P, Dray-Spira R, Duvivier C (2006). Predictors identified for losses to follow up among HIV-seropositive patients.. Journal of Clinical Epidemiology.

[24] Kaplan R, Orrell C, Zwane E, Bekker LG, Wood R (2008). Loss to follow-up and mortality among pregnant women referred to a community clinic for antiretroviral treatment.. Aids.

[25] McCoy D, Besser M, Visser R, Doherty T (2002). Interim findings on the National PMTCT pilot sites: Summary of lessons and recommendations. Durban: Health Systems Trust.. ftp://ftp.hst.org.za/pubs/pmtct/pmtctsummary.pdf.

[26] Tejiokem MC, Nerrienet E, Tene G, Menu E, Barre-Sinoussi F (2004). [Prevention of mother to child HIV-1 transmission (MTC) in Cameroon].. Med Mal Infect.

[27] Tchendjou P, Same-Ekobo C, Nga A, Tejiokem M, Kfutwah A (2010). Effectiveness of multidrug antiretroviral regimens to prevent mother-to-child transmission of HIV-1 in routine public health services in Cameroon.. PLoS One.

[28] Institut National de la Statistique, ORC-Macro (2004). Enquête démographique et de Santé, Cameroun.. http://www.measuredhs.com/pubs/pdf/FR163/FR163-CM04.pdf.

