# Supplementary material for: Feasibility of Early Infant Diagnosis of HIV in Resource-Limited Settings: The ANRS 12140-PEDIACAM Study in Cameroon
Source: PLoS One. 2011 Jul 19;6(7):e21840. doi: 10.1371/journal.pone.0021840 (PMC3139572; doi:10.1371/journal.pone.0021840)
Supplement: Table S1 — Univariate analysis: factors associated with incomplete HIV early infant diagnosis process: (ANRS 12140-Pediacam study, Cameroon, 2007–2009). (DOC) [file pone.0021840.s001.doc]

Table 1: Univariate analysis: factors associated with incomplete HIV early infant diagnosis process: (ANRS 12140-Pediacam study, Cameroon, 2007-2009)

| **Variables** | N | n | % | Crude Odds ratio (OR) [CI 95%] | p-value |
| --- | --- | --- | --- | --- | --- |
| **Total** | 1587 | 256 | 16.1 |  |  |
| **Mothers’ characteristics *(n, %)*** |  |  |  |  |  |
| Clinical sites *(1587, 100)* |  |  |  |  | 0.68 |
| Essos Hospital Center, Yaoundé | 392 | 62 | 15.8 | 1.0 (0.7-1.4) |  |
| Laquintinie Hospital, Douala | 466 | 81 | 17.4 | 1.1 (0.8-1.6) |  |
| Central Hospital / Mother and Child Center, Yaoundé | 729 | 113 | 15.5 | ref |  |
| Mothers' age at delivery *(1563, 98.5)* |  |  |  |  | 0.22 |
| <25 years | 314 | 58 | 18.5 | 1.4 (0.96-2.0) |  |
| 25-30 years | 560 | 86 | 15.4 | 1.1 (0.8-1.5) |  |
| >30 years | 689 | 98 | 14.2 | ref |  |
| Marital status *(1556, 98.0)* |  |  |  |  | 0.47 |
| Divorced or widow | 26 | 5 | 19.2 | 1.6 (0.6-4.3) |  |
| Single | 461 | 70 | 15.2 | 1.2 (0.8-1.7) |  |
| Cohabiting | 706 | 118 | 16.7 | 1.3 (0.9-1.9) |  |
| Married | 363 | 48 | 13.2 | ref |  |
| Mothers' level of education *(1550, 97.7)* |  |  |  |  | 0.05 |
| None or primary education | 295 | 59 | 20.0 | 1.6 (1.0-2.6) |  |
| Secondary | 1004 | 148 | 14.7 | 1.1 (0.8-1.7) |  |
| Higher education | 251 | 33 | 13.2 | ref |  |
| Mothers' professional activity *(1543, 97.2)* |  |  |  |  | 0.47 |
| Housewife or unemployed | 675 | 113 | 16.7 | 1.2 (0.9-1.6) |  |
| Training or student | 184 | 31 | 16.8 | 1.2 (0.8-1.9) |  |
| Paid activity | 684 | 99 | 14.5 | ref |  |
| Presence of a functional fridge at home *(1534, 96.7)* |  |  |  |  |  |
| No | 725 | 128 | 17.7 | 1.3 (1.0-1.7) | 0.05 |
| Yes | 809 | 113 | 14.0 | ref |  |
| Running water at home *(1539, 97.0)* |  |  |  |  |  |
| No | 688 | 113 | 16.4 | 0.9 (0.7-1.2) | 0.46 |
| Yes | 851 | 128 | 15.0 | ref |  |
| Electricity at home *(1539, 97.0)* |  |  |  |  |  |
| No | 36 | 7 | 19.4 | 0.8 (0.3-1.8) | 0.53 |
| Yes | 1503 | 234 | 15.6 | ref |  |
| Time of maternal HIV diagnosis *(1566, 98.7)* |  |  |  |  |  |
| Less than 3 months before delivery | 351 | 79 | 22.5 | 2.1 (1.5-3.0) | <10-3 |
| More than 3 months before delivery | 549 | 83 | 15.1 | 1.3 (0.9-1.8) |  |
| Before pregnancy | 666 | 81 | 12.2 | ref |  |
| Disclosure of maternal HIV serostatus to partner *(1406, 88.6)* |  |  |  |  | 0.03 |
| No | 267 | 52 | 19.5 | 1.5 (1.0-2.1) |  |
| Yes | 1139 | 160 | 14.1 | ref |  |
| Maternal knowledge of the fathers' HIV status *(1465, 92.3)* |  |  |  |  | 0.11 |
| Unknown | 587 | 101 | 17.2 | 1.5 (1.0-2.2) |  |
| HIV-negative | 516 | 81 | 15.7 | 1.3 (0.9-2.0) |  |
| HIV-positive | 362 | 44 | 12.2 | ref |  |
| Primipara *(1560, 98.3)* |  |  |  |  | 0.61 |
| Yes | 246 | 41 | 16.7 | 1.1 (0.8-1.6) |  |
| No | 1314 | 202 | 15.4 | ref |  |
| Number of antenatal visits *(1507, 95.0)* |  |  |  |  |  |
| ≤3 | 220 | 46 | 20.9 | 1.6 (1.1-2.2) | 0.02 |
| >3 | 1287 | 187 | 14.5 | ref |  |

*n: number of mother-infant pairs in each characteristic showing missing data related to the overall population*

**Table 1:** (continued)

| **Variables** | N | n | % | Crude Odds ratio (OR) [CI 95%] | p-value |
| --- | --- | --- | --- | --- | --- |
| **Total** | 1587 | 256 | 16.1 |  |  |
| **Mothers’ characteristics *(n, %)*** |  |  |  |  |  |
| History of any disease during pregnancy *(1527, 96.2)* |  |  |  |  |  |
| No | 925 | 151 | 16.3 | 1.2 (0.9-1.5) | 0.32 |
| Yes | 602 | 87 | 14.4 | ref |  |
| Number of living children *(1558, 98.2)* |  |  |  |  |  |
| More than one | 1044 | 165 | 15.8 | 1.1 (0.8-1.4) | 0.60 |
| one | 514 | 76 | 14.8 | ref |  |
| Mode of delivery *(1564, 98.6)* |  |  |  |  | 0.002 |
| Emergency C-section | 96 | 27 | 28.1 | 2.2 (1.4-3.6) |  |
| Elective C-section | 53 | 7 | 13.2 | 0.9 (0.4-2.0) |  |
| Vaginal | 1415 | 209 | 14.8 | ref |  |
| Sites of delivery *(1587, 100)* |  |  |  |  |  |
| At home or other health structure | 411 | 72 | 17.5 | 1.1 (0.8-1.5) | 0.37 |
| Pediacam study sites | 1176 | 184 | 15.6 | ref |  |
| Receipt of any ART for PMTCT *(1535, 96.7)* |  |  |  |  | <10-4 |
| No prophylaxis | 157 | 50 | 31.8 | 4.0 (2.6-6.3) |  |
| Short-course combined ARV | 790 | 124 | 15.7 | 1.6 (1.2-2.2) |  |
| HAART | 588 | 61 | 10.4 | ref |  |
| **Fathers’ characteristics *(n, %)*** |  |  |  |  |  |
| Fathers' level of education *(1380, 87.0)* |  |  |  |  | 0.29 |
| Mother did not know | 244 | 44 | 18.0 | 1.4 (0.9-2.1) |  |
| None or primary education | 126 | 15 | 11.9 | 0.8 (0.4-1.5) |  |
| Secondary | 607 | 100 | 16.5 | 1.2 (0.8-1.7) |  |
| Higher education | 403 | 56 | 13.9 | Ref |  |
| Fathers’ age at infant enrolment *(1401, 88.3)* |  |  |  |  | 0.30 |
| <35 years | 565 | 87 | 15.4 | 1.2 (0.8-1.7) |  |
| 35-39 years | 424 | 73 | 17.2 | 1.4 (0.9-2.0) |  |
| ≥40 years | 412 | 54 | 13.1 | ref |  |
| **Infants characteristics *(n, %)*** |  |  |  |  |  |
| Infant sex *(1587, 100)* |  |  |  |  |  |
| Male | 819 | 135 | 16.5 | 1.1 (0.8-1.4) | 0.69 |
| Female | 768 | 121 | 15.8 | ref |  |
| Birth weight *(1560, 98.3)* |  |  |  |  |  |
| <2500g | 148 | 29 | 19.6 | 1.4 (0.9-2.1) | 0.14 |
| ≥2500g | 1412 | 211 | 14.9 | ref |  |
| Feeding option *(1567, 98.7)* |  |  |  |  |  |
| Breast feeding | 170 | 29 | 17.1 | 1.1 (0.7-1.7) | 0.55 |
| Formula feeding | 1397 | 214 | 15.3 | ref |  |
| Receipt of any ART for PMTCT *(1551, 97.7)* |  |  |  |  |  |
| No | 37 | 7 | 18.9 | 1.3 (0.6-3.0) | 0.54 |
| Yes | 1514 | 231 | 15.3 | ref |  |
| Infant hospitalised at birth *(1520, 95.8)* |  |  |  |  |  |
| Yes | 176 | 35 | 19.9 | 1.4 (0.96-2.1) | 0.07 |
| No | 1344 | 198 | 14.7 | ref |  |
| Prematurity *(1564, 98.6)* |  |  |  |  |  |
| <37 weeks of pregnancy | 228 | 44 | 19.3 | 1.4 (0.9-2.0) | 0.09 |
| ≥37 weeks of pregnancy | 1336 | 199 | 14.9 | ref |  |
| Multiple birth *(1587, 100)* |  |  |  |  |  |
| Yes | 56 | 14 | 25.0 | 1.8 (0.9-3.3) | 0.07 |
| No | 1531 | 242 | 15.8 | ref |  |
