# Supplementary material for: Feasibility of Early Infant Diagnosis of HIV in Resource-Limited Settings: The ANRS 12140-PEDIACAM Study in Cameroon
Source: PLoS One. 2011 Jul 19;6(7):e21840. doi: 10.1371/journal.pone.0021840 (PMC3139572; doi:10.1371/journal.pone.0021840)
Supplement: Appendix S1 — The ANRS 12140-Pediacam Study Team. (DOC) [file pone.0021840.s002.doc]

**Appendix S1**

The **ANRS 12140-Pediacam Study Team** is constituted as follows:

**Primary investigators:** Prof Albert Faye (Hôpital Robert Debré/Univ. Paris 7, France) and Dr Mathurin Cyrille Tejiokem (Centre Pasteur du Cameroun, Yaoundé)

**Co-investigators:** Prof Françoise Barré-Sinoussi and Dr Daniel Scott (Unité de Régulations des Infections Rétrovirales, Institut Pasteur de Paris, France), Dr Frédéric Tangy (Laboratoire de Génomique virale et Vaccinantion, Institut Pasteur de Paris, France), Dr Laurence Baril (GSK BIO, Bruxelles, Belgique), Dr Josiane Warszawski (Equipe 4 : VIH et IST - INSERM U1018 (CESP)/Univ. Paris Sud 11, France), Prof Stéphane Blanche (Service d’Immunologie et Hématologie Pédiatrique, Hôpital Necker Enfants Malades, Paris, France), Dr Catherine Dollfus (Hôpital Trousseau, Paris, France), Dr Pascal Boisier and Dr Anfumbom Kfutwah (Centre Pasteur du Cameroun), Dr Ida Penda (Hôpital Laquintinie, Douala, Cameroun), Dr Georgette Guemkam and Dr Ateba Ndongo Francis (Centre Mère et Enfant de la Fondation Chantal Biya, Yaoundé, Cameroun), Dr Gisèle Chewa (Centre Hospitalier d’Essos, Yaoundé, Cameroun).

**Other members of the ANRS 12140 Pediacam team:** (by site and alphabetic order)

*Centre Pasteur du Cameroun:* Pascaline Maffo, Paul Alain Ngoupo, Félicité Owona, Dr Régis Pouillot, Dr Dominique Rousset, Dr Patrice Tchendjou, Martial Yonga

*Center Hospital Maternity/Mother and Child care Center in Yaoundé:* Jean Marie Ehongo, Serge Ela, Prof Robert Leke, Patricia Mbida, Dr Jean Audrey Ndongo, Jeanne Ngozi, Claire Ndongo, Dr Gilbert Tene, and Prof Paul Koki

*Essos Hospital Center in Yaoundé:* Marie Louise Belinga, Dieudonné Evoundou, Dr Annie Nga, Suzanne Nguen, Dr Suzie Tetang, Dr Guillaume Wamba and Dr Zeudja

*Laquintinie Hospital in Douala:* Julie Djene, Dr Nicaise Makwet, Dr Madeleine Mbangué, Mary Nfor, Aurore Ngo Sohna, Dr Ngwa, Dr Hermine Nyemb, Shiro Obedat, Dr Gérémie Solle and Dr Cassandre Tocko.
